# Placenta-specific epimutation at *H19*-DMR among common pregnancy complications: its frequency and effect on the expression patterns of *H19* and *IGF2*

**DOI:** 10.1186/s13148-019-0712-3

**Published:** 2019-08-01

**Authors:** Yuko Yamaguchi, Chiharu Tayama, Junko Tomikawa, Rina Akaishi, Hiromi Kamura, Kentaro Matsuoka, Norio Wake, Hisanori Minakami, Kiyoko Kato, Takahiro Yamada, Kazuhiko Nakabayashi, Kenichiro Hata

**Affiliations:** 1 Department of Maternal-Fetal Biology, Research Institute, National Center for Child Health and Development, Tokyo, 157-8535 Japan; 2 Department of Obstetrics and Gynecology, Graduate School of Medical Sciences, Kyushu University, Fukuoka, 812-8582 Japan; 3 Center of Maternal-Fetal, Neonatal and Reproductive Medicine, National Center for Child Health and Development, Tokyo, 157-8535 Japan; 4 Department of Pathology, National Center for Child Health and Development, Tokyo, 157-8535 Japan; 5 Department of Obstetrics and Gynecology, Hokkaido University Graduate School of Medicine, Sapporo, 060-8638 Japan; 6 Clinical Genetics Unit, Kyoto University Hospital, Kyoto, 606-8507 Japan; 7 Present Address: Department of Pathology, Dokkyo Medical University, Saitama Medical Center, Koshigaya, Japan

**Keywords:** Genomic imprinting, DNA methylation, Epimutation, Placenta, *H19*, *IGF2*, Fetal growth restriction, Pregnancy-induced hypertension, Hypertensive disorder of pregnancy

## Abstract

**Background:**

*H19* and *IGF2* genes are imprinted and involved in regulating fetal and placental growth. The *H19* differentially methylated region (DMR) is paternally methylated and maternally unmethylated and regulates the imprinted expression of *H19* and *IGF2*. Epimutation at the *H19*-DMR in humans results in congenital growth disorders, Beckwith-Wiedemann and Silver-Russell syndromes, when erroneously its maternal allele becomes methylated and its paternal allele becomes unmethylated, respectively. Although *H19* and *IGF2* have been assessed for their involvement in pregnancy complications including fetal growth restriction (FGR) and pregnancy-induced hypertension (PIH)/hypertensive disorder of pregnancy (HDP) intensively in the last decade, it is still not established whether epimutation at the *H19*-DMR in the placenta results in pathogenic conditions in pregnancy. We aimed to assess the frequency of *H19*-DMR epimutation and its effects on the allelic expression patterns of *H19* and *IGF2* genes among normal and abnormal pregnancy cases.

**Results:**

We enrolled two independently collected sets of placenta samples from normal pregnancies as controls and common pregnancy complications, FGR and PIH (HDP). The first set consisted of 39 controls and 140 FGR and/or PIH cases, and the second set consisted of 29 controls and 62 cases. For these samples, we initially screened for DNA methylation changes at *H19*-DMR and *IGF2*-DMRs by combined bisulfite restriction analysis, and further analyzed cases with methylation changes for their allelic methylation and expression patterns. We identified one case each of FGR and PIH showing hypomethylation of *H19*-DMR and *IGF2*-DMRs only in the placenta, but not in cord blood, from the first case/control set. For the PIH case, we were able to determine the allelic expression pattern of *H19* to be biallelically expressed and the *H19/IGF2* expression ratio to be highly elevated compared to controls. We also identified a PIH case with hypomethylation at *H19*-DMR and *IGF2*-DMRs in the placenta from the second case/control set.

**Conclusions:**

Placental epimutation at *H19*-DMR was observed among common pregnancy complication cases at the frequency of 1.5% (3 out of 202 cases examined), but not in 68 normal pregnancy cases examined. Alteration of *H19/IGF2* expression patterns due to hypomethylation of *H19*-DMR may have been involved in the pathogenesis of pregnancy complications in these cases.

**Electronic supplementary material:**

The online version of this article (10.1186/s13148-019-0712-3) contains supplementary material, which is available to authorized users.

## Background

Genome imprinting is an epigenetic mechanism whereby only one of the two parental alleles is expressed, and has been known to be critical for proper development of the fetus and the placenta. Imprinted genes tend to be clustered in the genome. Allelic expression patterns of a cluster of imprinted genes have been shown to be regulated in *cis* by an imprinting control region (ICR). ICRs overlap with a differentially methylated region (DMR) that exhibits parental allele-specific DNA methylation inherited from gametes (sperm and oocyte) and maintained throughout subsequent development. ICRs (germline DMRs) are essential in establishing additional somatic DMRs and allele-specific histone modifications within imprinted domains [1]. *H19* and *IGF2* genes are imprinted (maternally and paternally expressed, respectively) and regulated by *H19*-DMR, the ICR for these genes being located at 2.5 kb upstream of the *H19* promoter region. *H19* and *IGF2* are known to be crucial for fetal and postnatal growth [2]. Abnormal DNA methylation (epimutation) at *H19*-DMR occurring in gametes or early development leads to specific imprinting disorders. Hypermethylation at *H19*-DMR causes Beckwith-Wiedemann syndrome (BWS), a congenital overgrowth syndrome occasionally associated with embryonal tumors (such as Wilms tumor, hepatoblastoma, adrenal carcinoma, and neuroblastoma) [3, 4]. Hypomethylation at *H19*-DMR causes Silver-Russell syndrome (SRS), which is characterized by prenatal and postnatal growth restriction with additional dysmorphic features [5]. Increased and decreased expression levels of the *IGF2* gene encoding insulin-like growth factor 2 due to ICR epimutation are considered to be the leading causes of BWS and SRS, respectively [3, 5].

Previous studies using mouse models have shown the involvement of *H19* and *Igf2* in the development and functions of the placenta. *H19* deletion in mice has been shown to result in increased *Igf2* expression and fetal/placental overgrowth [6, 7] and reduced nutrient transport capacity [8]. *Igf2* deletion in mice is shown to result in fetal/placental growth restriction [9, 10] and altered diffusional exchange properties [11]. These lines of evidence from mouse studies suggested that imprinting defects at the *H19/IGF2* gene cluster in the human placenta could also cause placental dysfunction and be involved in pregnancy complications. Accordingly, the *H19-IGF2* imprinted gene cluster has been intensively characterized by itself or together with other key imprinted genes in the placentas from common pregnancy complications such as FGR and PIH in the last decade [12–18]. Despite past efforts to gain knowledge of its etiological aspect, the cause of PIH is still unclear [19]. The etiologies of FGR are considered to be multifactorial (maternal, fetal, placental, and environmental) [20]. Therefore, the etiologies of all PIH cases and the majority of FGR cases are unknown. These pregnancy complications are considered to be heterogeneous conditions for which a variety of genetic and environmental factors contribute to their onset [21]. It should be noted that PIH has been recently renamed as hypertensive disorder of pregnancy (HDP). Gene expression and DNA methylation properties assessed in the studies of *H19-IGF2* locus in FGR and PIH cases [12–18] include gene expression levels and loss of imprinting (LOI) frequencies of *H19* and *IGF2*, and DNA methylation levels of *H19* germline DMR, *H19* promoter, and *IGF2* somatic DMRs in the placentas with or without pregnancy complications. The extent of consistency among the results from independent studies varies depending on the property examined as described below.

LOI, biallelic expression of an imprinted gene due to the reactivation of the normally repressed allele, of *H19* at the third trimester placentas was initially reported to occur only in PIH but not in normal pregnancy cases [14]. However, in later studies [16, 18], LOI of *H19* in the third trimester and full-term placentas was detected regardless of the presence/absence of obstetric complications. LOI of *IGF2* in the placentas was frequently observed (5 out of 16 cases) in one study [13], but not in another study (none in 92 cases) [18]. It should be noted that the criteria for LOI (or relaxation of imprinting, ROI) were significantly different between these two studies (> 3% and > 25%, respectively). In these LOI studies [13, 14, 18], LOI of *H19* and *IGF2* in the placenta did not correlate with the gene expression levels. Gene expression levels of *H19* and *IGF2* were shown to be correlated (with statistical significance) both in the placenta (*n* = 36) and the cord blood (*n* = 100) in one study [18], although such a correlation was not observed in the placenta (*n* = 34) in another study [12]. Further evaluation of the gene expression patterns of *H19* and *IGF2* in the placenta is important to validate these previous findings.

Whereas two studies [15, 17] reported statistically significantly lower levels of DNA methylation of *H19*-DMR in FGR placentas compared to normal placentas, such association was not detected by another study [12]. Small sample sizes in these studies may explain the discrepant results. Whereas Guo et al. [12] identified a small for gestational age (SGA) case exhibiting hypomethylation at *H19*-DMR accompanied with the biallelic expression of *H19* in the placenta among the 22 SGA cases, Tabano et al. [16] did not find such *H19*-DMR epimutation cases among the placenta samples of the 66 FGR cases. Additional and larger-scale studies may help further establish the roles of the *H19/IGF2* gene cluster in the pathogenesis of pregnancy complications.

In this study, we aimed to determine whether and how frequently epimutation at *H19*-DMR occurs in the placentas from common pregnancy complications, FGR and PIH, and also assess some of the previously suggested features of gene expression and methylation patterns at the *H19/IGF2* imprinted gene cluster in our sample collections.

## Results

### Identification of FGR and PIH placentas with H19-DMR hypomethylation

**Fig. 1:**
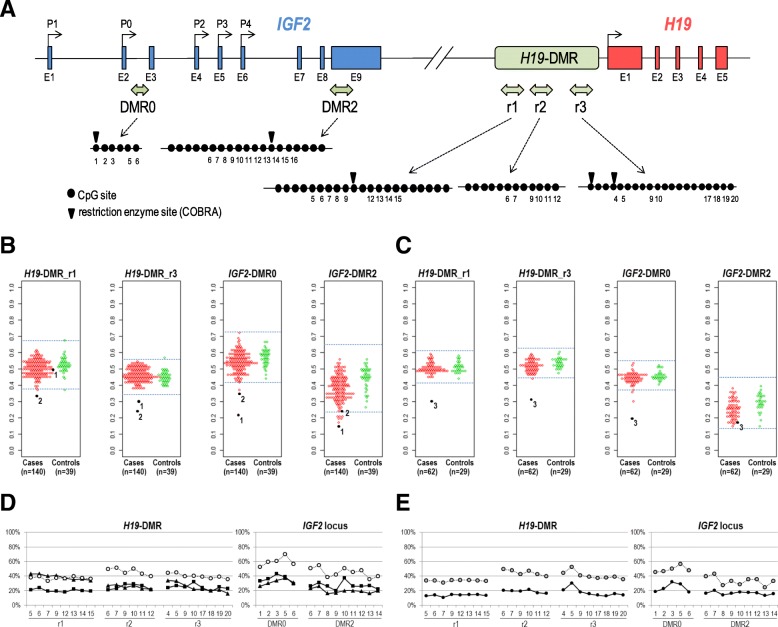
DNA methylation analyses for *H19*-DMR and *IGF2*-DMRs by COBRA and pyrosequencing assays. **a** Location of CpG sites within *H19*-DMR, *IGF2*-DMR0, and *IGF2*-DMR2 analyzed. Exons of *IGF2* and *H19* genes are shown as blue and red squares, respectively. Transcriptional start sites of *IGF2* (P0-P4) and *H19* are indicated by arrows. The approximate positions of the genomic intervals subjected to DNA methylation analyses within *H19*-DMR, *IGF2*-DMR0, and *IGF2*-DMR2 are shown by light green two-headed arrows. The genomic intervals of *IGF2*-DMR0 and *IGF2*-DMR2 were described previously [17]. Closed circles represent CpG sites included in the bisulfite-PCR amplicons subjected to COBRA, pyrosequencing, and bisulfite sequencing analyses. Downward arrowheads in the r1 and r3 subregions within *H19*-DMR indicate TaqI sites used in COBRA assays. The CpG sites whose methylation level was assessed by pyrosequencing are numbered underneath the closed circles. **b**, **c** DNA methylation levels of *H19*-DMR (r1 and r3 subregions) and *IGF2*-DMRs in FGR and/or *PIH* placentas determined by COBRA. The methylation levels were measured for chorionic plate of case group I (*n* = 140) and control group I (*n* = 39) (**b**) and for chorionic villi of case group II (*n* = 62) and control group II (*n* = 27) (**c**). Methylation levels corresponding to ± 3SD of the mean of control samples, our criterion for aberrant DNA methylation, are shown by blue horizontal dashed lines in each panel. The dots for cases 1 and 2 (in **b**) and for case 3 (in **c**) are shown in black and with the corresponding number. **d**, **e** Methylation levels at *H19*-DMR and *IGF2*-DMRs in the placenta of cases 1–3 examined by pyrosequencing. The methylation levels in case 1 (closed triangle), case 2 (closed rectangle), and the average of five controls from the control set I (chorionic plate samples) (open circle) are shown in **d**. The methylation levels in case 3 (black circle) and the average of five controls from the control set II (chorionic villi samples) (grey circle) in *H19*-DMR, *IGF2*-DMR0, and *IGF2*-DMR2 are shown in **e**. The numbers for CpG sites shown underneath the plots (**d**, **e**) correspond to those depicted in **a**

To screen for cases with DNA methylation abnormality at *H19*-DMR among FGR and/or PIH placentas, we measured the DNA methylation levels of *H19*-DMR in placenta samples by combined bisulfite restriction analysis (COBRA). We analyzed two subregions (r1 and r3 in Fig. 1a) within *H19*-DMR. Both subregions have previously been shown to be differentially methylated (paternally methylated) in human fetal tissues [22, 23]. The r1 region includes the sixth CTCF-binding site, which has been suggested to be a key regulatory domain for the imprinted expression of *H19* and *IGF2* genes [22]. The r3 region corresponds to a promoter-proximal region (H421), which was previously reported to be paternally methylated [23]. We initially subjected a total of 140 cases and 39 controls (case/control set I, Table 1) to COBRA. Genomic DNA was isolated from the chorionic plate of placentas for the case/control set I. The mean and standard deviation (SD) of the methylation levels of the 39 control samples were 52.2% (SD 4.8%) and 44.8% (SD 3.6%) for *H19*-DMR_r1 and *H19*-DMR_r3, respectively. We considered methylation levels over/under the range of ± 3SD of the mean as aberrant methylation. Two cases (designated as case 1 and case 2 hereafter) were found to be aberrantly hypomethylated at *H19*-DMR. Case 1, an FGR case, was hypomethylated at *H19*-DMR_r3 (30.1%, − 4.1SD), but not at *H19*-DMR_r1 (50.1%, − 0.4SD). Case 2, a PIH case, was hypomethylated at both *H19*-DMR_r1 (33.2%, –4.0SD) and *H19*-DMR_r3 (24.5%, − 5.5SD) (Fig. 1b). To screen for additional cases with aberrant methylation at *H19*-DMR, we conducted COBRA for another set of cases and controls, which were collected independently from the first case/control set. The second case/control set consisted of 62 FGR and/or PIH cases and 29 controls (case/control set II in Table 1). Genomic DNA was isolated from chorionic villi of the placentas of the case/control set II. The means and SD of the methylation levels in control group II were 51.2% (SD 4.1%) and 52.7% (SD 2.8%) for *H19*-DMR_r1 and *H19*-DMR_r3, respectively. One PIH case (designated as case 3) was identified to be hypomethylated at *H19*-DMR_r1 (29.4%, − 5.3SD) and *H19*-DMR_r3 (31.4%, − 7.6SD) (Fig. 1c). We validated the observed hypomethylation at *H19*-DMR in cases 1–3 (Fig. 1b, c) by pyrosequencing analysis (for 23 CpG sites within *H19*-DMR, 6 CpG sites within *IGF2*-DMR0, and 11 CpG sites within *IGF2*-DMR2 as depicted in Fig. 1a). Consistent with the results obtained by COBRA assays, the r1 subregion was confirmed to be hypomethylated in case 2 and case 3, and the r2 and r3 subregions were hypomethylated in three cases (Fig. 1d, e). The methylation levels at *IGF2*-DMR0 and *IGF2*-DMR2 were also found to be lower than the mean of the controls in all cases (Fig. 1d, e). For case 2, we also analyzed a second chorionic plate sample and observed similar DNA methylation levels. These results of hypomethylation of *H19*-DMR accompanied with hypomethylation of *IGF2*-DMRs suggest the regulatory role of *H19*-DMR in the somatic establishment of differential methylation at *IGF2*-DMR0 and *IGF2*-DMR2 in the human placenta, as has been shown for the mouse *H19/Igf2* imprinted domain [24]. Summarizing the above, we identified three cases with aberrant DNA hypomethylation at *H19*-DMR out of 202 placentas from FGR and/or PIH cases.

**Table 1:** Summary of clinical information for case and control groups enrolled in this study

Case/control group (or case #)	Average ± SD			
	Gestational age (week)	Fetal weight (g)	Placental weight (g)	Fetal weight/placental weight
Set I				
Control group I ^1)^ (*n* = 39)	38.8 ± 1.3	3002 ± 407	506 ± 129	6.2 ± 1.9
Case group I ^1)^ (FGR and/or PIH, *n* = 140)	35.5 ± 4.4	1793 ± 596	277 ± 89	6.5 ± 1.7
(statistical test *p* value)	(1.59 × 10^−8^)	(1.08 × 10^−25^)	(8.91 × 10^−23^)	(0.289)
Case 1 (FGR)	30	724	115	6.3
Case 2 (PIH)	30	1376	213	6.5
Set II				
Control group II ^2)^ (*n* = 29)	39.1 ± 1.3	3059 ± 311	601 ± 100	5.2 ± 0.8
Case group II ^2)^ (FGR and/or PIH, *n* = 62)	35.7 ± 2.6	2017 ± 545	456 ± 127	4.5 ± 1.0
(statistical test *p* value)	(3.81 × 10^−10^)	(1.00 × 10^−25^)	(3.42 × 10^−7^)	(0.0016)
Case 3 (PIH)	33	1628	420	3.9

^1)^Chorionic plate tissues were collected for all cases and controls. Cord blood samples were collected for subset of cases and controls

^2)^Only chorionic villi were collected for all cases and controls

### Correlation and association analyses of DNA methylation levels among loci and tissues and with pregnancy complications

We assessed correlation of DNA methylation levels of *H19*-DMRs and *IGF2*-DMRs. Within a tissue, *H19*-DMR_r1 and *H19*-DMR_r3 were most highly correlated in the cord blood (correlation coefficient *r* = 0.80) and moderately correlated (*r* = 0.50) in the placentas (chorionic plate and villi) (Additional file 1: Figure S1). *IGF2*-DMR0 and *IGF2*-DMR2 were moderately and weakly correlated in the chorionic plate (*r* = 0.47) and the cord blood (*r* = 0.26), respectively, but not in chorionic villi (*r* = 0.15) (Additional file 1: Figure S1). DNA methylation levels of *H19*-DMR and *IGF2*-DMRs were moderately correlated only in chorionic villi (*r* = 0.41 for *H19*-DMR_r3 vs *IFG2*-DMR0), but not in chorionic plate or cord blood. Between the chorionic plate and the cord blood, whereas DNA methylation levels of *H19*-DMR were not correlated (*r* = 0.18 for r1 and *r* = 0.20 for r3), those of *IGF2*-DMRs were moderately and weakly correlated (*r* = 0.39 for DMR0 and *r* = 0.26 for DMR2) (Additional file 1: Figure S1).

**Fig. 2:**
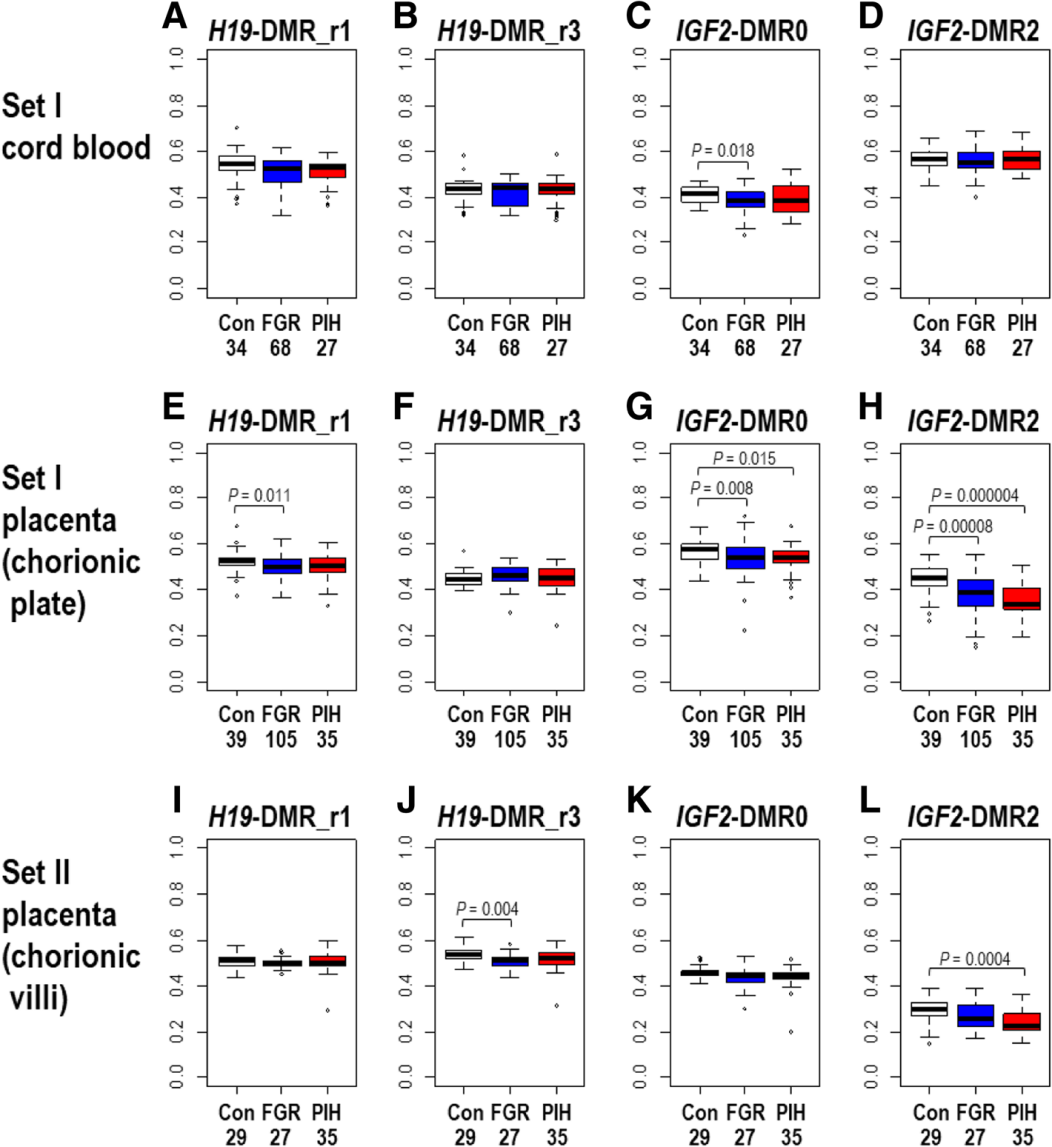
**a**–**l** Comparison of methylation levels of *H19*-DMR and *IGF2*-DMRs in FGR, PIH, and control (Con) groups. FGR cases without PIH phenotype and PIH cases with or without FGR phenotype are regarded as FGR and PIH groups, respectively, in this analysis. The numbers of individuals in each group are shown underneath each panel. Statistical significance of difference in DNA methylation levels between FGR or PIH group and a control group was assessed by the Wilcoxon signed-rank test (R version 3.4.4) (Additional file 3: Table S1). *P* values are shown for the pairs of comparisons for which statistical significance (*p* < 0.05) was detected

We also assessed the differences in DNA methylation levels in the FGR and PIH groups compared to controls (Fig. 2 and Additional file 3: Table S1). In the cord blood samples (of set I), the methylation levels of *H19*-DMR were not significantly different in 105 FGR cases without PIH phenotype or in 29 PIH cases with or without FGR phenotype compared with 39 controls. On the other hand, in the placental samples, the methylation levels of both *H19*-DMR_r1 and *H19*-DMR_r3 were statistically significantly lower in FGR cases compared to the controls (*p* = 0.011 in Fig. 2e and *p* = 0.004 in Fig. 2j). These results of the association of lower methylation levels with the FGR phenotype are in agreement with a previous report by Bourque et al. [15]. The methylation levels of *IGF2*-DMR0 were statistically significantly lower in FGR cases in both the cord blood and placentas and in PIH cases only in the placentas of set I (Fig. 2c, g). Whereas the statistical significance of the abovementioned lower levels of methylation at *H19*-DMR and *IGF2*-DMR0 tended to be marginal to the statistical threshold (*p* = 0.05), methylation levels at *IGF2*-DMR2 were more significantly lower both in FGR and PIH cases in the placentas of set I (Fig. 2h, *p* = 0.00008 and *p* = 0.000004, respectively) and in PIH cases in the placentas of set II (Fig. 2l, *p =* 0.0004). These results suggest that the DNA methylation status of *IGF2*-DMR2 may be used as a placental biomarker for pregnancy complication phenotypes (in particular, PIH) and placental dysfunctions.

### Hypomethylation at H19-DMR in FGR and PIH cases is accompanied with hypomethylation of IGF2-DMRs and is confined to the placenta

**Fig. 3:**
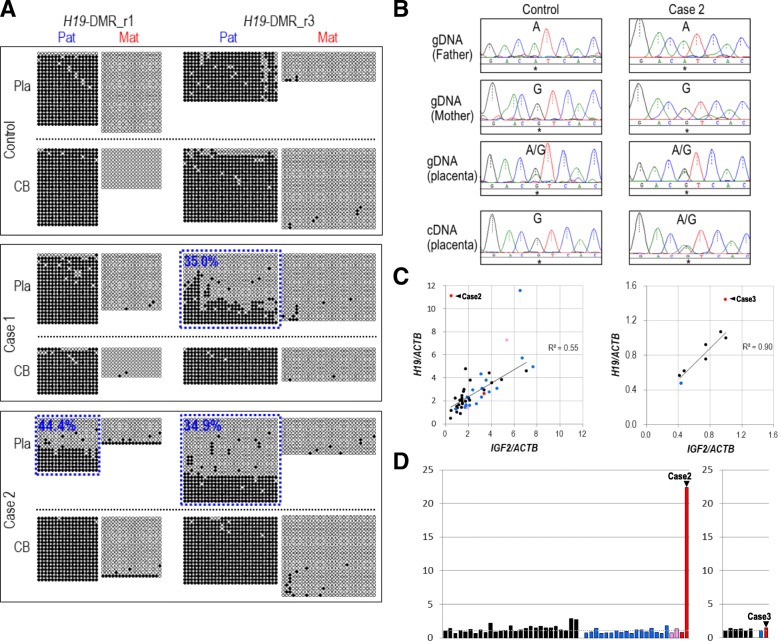
Allelic DNA methylation and expression analyses and quantitative expression analysis of *H19* and *IGF2* for cases 1 to 3. **a** Placenta-specific paternal hypomethylation at *H19*-DMR in case 1 and case 2 revealed by clone-based bisulfite sequencing with allelic discrimination. Open and closed circles represent unmethylated and methylated cytosines, respectively. Each row of circles corresponds to an individual clone sequenced. Pla and CB denote placenta (chorionic plate) and cord blood, respectively. Parental alleles were distinguished based on the genotypes of rs2107425 and rs10732516 for the r1 subregion and of rs2251312 and rs568497589 for the r3 subregion. Cases and their parents were genotyped for four SNPs to find informative ones to distinguish parental alleles. **b** Biallelic expression of *H19* in the chorionic plate of case 2. The upper sequencing electropherograms show the genotype at rs2067051 (an exonic A/G SNP within the *H19* gene) in case 2, a control chorionic plate sample (from the control set I), and their parents. The bottom electropherograms show the sequencing results of RT-PCR amplicons spanning rs2067051. The nucleotide position corresponding to rs2067051 is marked by an asterisk in each panel. **c** Intra-individual correlation of expression levels of *H19* and *IGF2* in placenta. Normalized expression levels of *H19* and *IGF2* relative to those of a control are plotted for a subset of samples from the case/control set I (left) and from the case/control set II (right). The numbers of cases and controls are 27 controls (black), 17 FGR cases (blue), 2 FGR/PIH cases (pink), and 2 PIH cases including case 2 (red) from the case/control set I, and 6 controls, a FGR case, and a PIH case (case 3) from the case/control set II. Normalized expression levels were determined by the delta-delta Ct method using *ACTB* as a normalization control. Coefficient of determination calculated by Excel is shown in each plot (case 2 and case 3 were excluded from the calculation). **d** Relative expression ratios of *H19/IGF2* in chorionic plate samples from the case/control set I (left) and in chorionic villi samples from the case/control set II (right). The expression ratios of *H19* and *IGF2* are shown for controls (*n* = 27, black), FGR cases (*n* = 17, blue), PIH/FGR cases (*n* = 2, pink), and PIH cases (*n* = 2 including case 2, red) from the case/control set I (left) and for controls (*n* = 6), a FGR case and a PIH case (Case 3) from the case/control set II. Ratios relative to one control sample shown leftmost are shown

We subsequently aimed to determine the allelic methylation patterns of *H19*-DMR in the placenta and the cord blood of cases 1–3 by bisulfite sequencing. We screened for heterozygous SNPs within *H19*-DMR to distinguish paternal and maternal alleles and identified such SNPs for cases 1 and 2. Case 3 was excluded from the allelic bisulfite sequencing analysis due to the absence of heterozygous SNPs within *H19*-DMR. In a sample from control group I, the paternal allele was nearly completely methylated (100% methylated) and the maternal allele was (nearly) completely unmethylated in both regions (r1 and r3) examined in the placenta (chorionic plate) as well as in the corresponding cord blood (Fig. 3a). In case 1, paternal hypomethylation was observed in the r3 region but not in the r1 region in the placenta. In case 2, paternal hypomethylation was observed in both regions in the placenta. These patterns of paternal hypomethylation in the placenta of case 1 and case 2 are well consistent with the results of COBRA (Fig. 1b) and pyrosequencing analysis (Fig. 1d). However, in the cord blood of case 1 and case 2, the paternal allele was nearly completely methylated (Fig. 3a). These results demonstrate that hypomethylation at *H19*-DMR in case 1 and case 2 occurred only in the placenta, but not in the fetus. The observed partial loss of paternal methylation at *H19*-DMR in these cases (34.9 to 44.4% of paternal methylation retained) suggests the possibility of epigenetic mosaicism at *H19*-DMR in these placentas.

### Biallelic expression of H19 in the placenta with H19-DMR hypomethylation

We tried to determine the allelic expression pattern of *H19* in the placentas of cases 1–3. However, due to the unavailability of total RNA for case 1 and due to the absence of heterozygous SNPs in the exonic regions of *H19* in case 3, we were able to assess the allelic expression pattern of *H19* only for case 2. We carried out direct sequencing of RT-PCR amplicons that include rs2067051 (a transcribed SNP at the *H19* locus). Whereas *H19* was found to be exclusively expressed from the maternal (G) allele in a control chorionic plate sample, it was apparently biallelically expressed (A/G) in the chorionic plate of case 2 (Fig. 3b). Such a loss of imprinting of *H19* was not observed in 20 additional control placenta samples with a normal level of DNA methylation at *H19*-DMR (data not shown). These results suggest that paternal hypomethylation at *H19*-DMR led to the expression of *H19* from its paternal allele, which is normally silent. We could not assess the allelic expression pattern of *IGF2* because no heterozygous SNP was found in the exons of *IGF2* in cases 1–3.

### Expression levels of H19 and IGF2 in the placentas with H19-DMR epimutation (cases 2 and 3)

Hypomethylation at *H19*-DMR on the paternal allele is expected to result in the expression of *H19* from the normally repressed paternal allele (as was confirmed in the placenta of case 2 (Fig. 3b)) and the suppression of *IGF2* expression [25, 26]. To validate these expected expression patterns, we quantitatively measured the expression levels of *H19* and *IGF2* in our placenta samples. Consistent with previous reports [12, 18], the placental expression levels of *H19* and *IGF2* were diverse among samples irrespective of the presence/absence of pregnancy complications. We observed positive correlations between the intra-individual expression levels of *H19* and *IGF2* in both sets of placenta samples (chorionic plate samples from case/control set I [*n* = 27, *R*^2^ = 0.55] and chorionic villi samples from case/control set II [*n* = 7, *R*^2^ = 0.90]) (Fig. 3c). We could not include case 1 in this analysis due to the unavailability of its RNA as aforementioned. It should be noted that the correlation coefficients were calculated for only the samples without *H19*-DMR hypomethylation (i.e., case 2 and case 3 were excluded). In case 2, the *H19/IGF2* ratio was 22.3, which was extraordinarily higher than those of the control chorionic plate samples (*n* = 27) and the cases without *H19*-DMR epimutation (*n* = 20) (ratios ranging from 0.6 to 2.7) (Fig. 2d, left), suggesting the increase of *H19* expression due to its expression from the normally repressed paternal allele and the resultant reciprocal decrease in *IGF2* expression from the paternal allele. However, the *H19/IGF2* ratio in case 3 was similar to those in the control chorionic villi samples (*n* = 6) examined (Fig. 3d, right).

### Absence of copy number alteration at H19/IGF2 locus suggests epimutation as a cause of H19-DMR hypomethylation

Confined placental mosaicism (CPM), the presence of trisomic cells predominantly in the placenta, was recently shown to be present in approximately 10% of FGR cases [27]. In the situation of confined placental trisomy of chromosome 11 due to excess of a maternal copy, the overall methylation levels of *H19*-DMR and *IGF2*-DMRs, which are paternally methylated, are expected to be lower (one of three copies is methylated) than those in the normal diploid placenta (one of two copies is methylated). Duplication of a genomic segment containing maternally derived *H19* and *IGF2* genes would also result in lower methylation levels of *H19*-DMR and *IGF2-*DMRs. Therefore, when only the overall methylation level is available, hypomethylation of *H19*-DMR could be interpreted in the following two ways: as a consequence of loss of methylation on the paternal allele and as a consequence of copy number change of the *H19* locus. To further evaluate whether hypomethylation at *H19*-DMR in the placentas of cases 1–3 is due to epimutation, we carried out chromosomal copy number analysis for the placental genomic DNA samples as well as available parental blood genomic DNA samples. In all three cases, we detected neither copy number changes at *H19/IGF2* locus nor aneuploidy of chromosome 11 (Additional file 2: Figure S3). We also confirmed the biparental inheritance of chromosome 11 in all three cases by comparing SNP genotypes between each placenta and corresponding parent(s). These results further suggest that hypomethylation of *H19*-DMR observed in the placentas of cases 1–3 is due to epigenetic alteration, but not copy number alteration.

### DNA methylation analysis of other imprinted DMRs and LINE1 by COBRA

To gain further insight into possible mechanisms underlying the epimutation at *H19*-DMR in the placentas of cases 1–3, we examined the methylation levels of 16 imprinted DMRs (other than *H19*-DMR) and LINE1 repetitive elements in the placentas of cases 1–3 and controls by COBRA assays. Considering the gestational weeks of cases 1–3 (30, 30, and 33 weeks, respectively, Table 1), we chose control samples both from preterm birth (24–34 weeks) and normal-term birth (37–40 weeks). At *H19*-DMR (the r1 and r3 subregions), the distribution of methylation levels was similar between normal-term controls and preterm controls in both of the chorionic plate sample set (Additional file 1: Figure S2A) and the chorion sample set (Additional file 1: Figure S2B). The methylation levels of other DMRs and LINE1 elements in the placentas of cases 1–3 were found to be within the normal range (mean ± 3SD of control data) with the following few exceptions: methylation levels of *ARHI*-DMR in case 2 and of *ZAC*-DMR in case 3 were slightly out of the normal range (0.4% and 1.0% lower than “average − 3SD”). However, their extent of hypomethylation is much smaller than those observed for *H19*-DMR (e.g., 17.3% lower than “average − 3SD” at *H19*-DMR_r1 in case 2). These results suggest that the placental epimutation in cases 1–3 occurred locally at *H19*-DMR, but not in a genome-wide manner.

## Discussion

In this study, we demonstrated that epimutation at *H19*-DMR could occur in a placenta-specific manner. Among 202 FGR and/or PIH cases in total, we identified three cases, one FGR case (case 1) and two PIH cases (cases 2 and 3), showing *H19*-DMR hypomethylation in the placenta. For cases 1 and 2, we also revealed that the hypomethylation on the paternal allele of *H19*-DMR is specific to placenta, but not observed in cord blood (Fig. 3a). *IGF2* DMRs were also hypomethylated in the placentas of cases 1–3. We demonstrated that *H19* was biallelically expressed and the relative *H19/IGF2* expression ratio was 22.3 times higher in the placenta of case 2 than that of the control. In a precedent study conducting methylation analysis of *H19*-DMR in the placentas, Guo et al. [12] examined 22 SGA cases and identified one hypomethylation case accompanied with *H19* biallelic expression, but without the elevation in *H19/IGF2* expression ratio. It is not explicitly mentioned whether the authors examined the methylation status of *H19*-DMR in the corresponding cord blood. Two FGR cases in Koukoura et al. [17] and one FGR case in Bourque et al. [15] also showed extremely low (≈ 0%) and notably low (< 25%) methylation levels at *H19*-DMR in the placenta, but they were not characterized further. On the other hand, Tabano et al. [16] did not find any *H19*-DMR epimutation cases among 66 FGR placentas [13]. It should be also mentioned that Rancourt et al. [18] examined the DNA methylation levels of *H19* DMR in 114 placentas from normal pregnancy cases and identified one case showing hypermethylation at *H19*-DMR (presumably due to the gain of methylation on the maternal allele) without apparent alterations of *H19* and *IGF2* gene expression levels. Considering the results of these studies and ours together, extreme hypomethylation (i.e., epimutation) at *H19*-DMR in the placenta has been observed only in the pregnancies with complications. We clearly demonstrated that epimutation at *H19*-DMR could occur in a placenta-specific manner and is accompanied with hypomethylation at *IGF2* DMRs. The frequency of *H19*-DMR hypomethylation in the placenta was 1.5% (3/202) among the FGR and/or PIH pregnancy cases.

It is well established that germline epimutations at *H19*-DMR cause two growth disorders with opposite phenotypes: an overgrowth disorder, Beckwith-Wiedemann syndrome (when hypermethylated), and a growth retardation disorder, Silver-Russell syndrome (when hypomethylated). Increased and decreased expression levels of *IGF2* due to epimutation are considered to be the primary cause of BWS and SRS, respectively [3, 5]. In contrast, it remains unknown whether the placenta-specific epimutation at *H19*-DMR identified in this study and by Guo et al. [12] is responsible for the associated phenotypes such as FGR (SGA) and PIH. Considering that a variety of genetic and environmental influences can contribute to the risk of these common pregnancy complications [21], the low frequency (1.5%) of epimutation at *H19*-DMR does not necessarily diminish its potential significance. PIH and FGR are considered to be multifactorial diseases, and their causal genetic and /or epigenetic factors are scarcely known [19, 20]. In such a situation, it is important to identify candidate genes involved in their etiologies even for a subset of cases. Recent independent studies have revealed critical functions of *H19* lincRNA as a key regulator in multiple physiological processes and cell types [28–31]. Such regulatory roles of *H19* lincRNA in various tissues also suggest a potential impact of the dysregulation of *H19* expression in the placenta. However, evidence is currently lacking to link the observed epimutation with placental dysfunction and FGR/PIH phenotypes. Recently established CRISPR/dCas9-based epigenome editing technologies [32] could be applied to create cell line and mouse models of placenta-specific *H19*-DMR epimutation to elucidate its functional outcomes at the cellular and organ levels.

Consistent with a previous study [18], we observed the correlation of *H19* and *IGF2* expression levels in placentas (Fig. 3c, d). Furthermore, we revealed an extreme elevation of *H19/IGF2* expression ratio in case 2 with placenta-specific epimutation at *H19*-DMR, implying an increase of *H19* expression and decrease of *IGF2* expression due to the disruption of imprinted regulation. These results suggest the possibility that *H19* and *IGF2* expression levels vary among the human population presumably due to genetic diversity, that the appropriate balance of *H19* and *IGF2* expression levels is required for normal development, and that the extreme disturbance of the *H19/IGF2* expression ratio in the placenta of case 2 may have impaired placental development and functions. However, it should be also mentioned that this dysregulated pattern of *H19* and *IGF2* expression was not observed in case 3 in our study and the *H19*-DMR epimutation case identified by Guo et al. [12]. It is plausible that the outcome of *H19*-DMR epimutation on gene expression of *H19* and *IGF2* differs between chorionic plate (case 2) and chorionic villi (case 3 and #8928 in Guo et al. [12]) and also differs depending on the developmental stages. Correlations of the expression levels of imprinted genes such as *IGF2* and *IGF2R* and fetal weight have been detected in a manner dependent of the developmental stages of placenta [33].

Yamazawa et al. demonstrated that *H19*-DMR hypomethylation affects the imprinted expression of *H19* and *IGF2* in both fetus and placenta in several SRS cases [34], strongly suggesting the germline origin of *H19*-DMR epimutation in the SRS cases examined. On the other hand, we have previously reported two BWS cases whose methylation levels of *H19*-DMR were discordant in embryo-derived somatic tissues (blood and skin) and placenta, suggesting that the aberrant gain of methylation on the maternally derived allele occurred after implantation in these cases [35]. The extent of gain of methylation at *H19*-DMR was higher in blood and skin than that in the placenta. The epimutation at *H19*-DMR was specific to the placenta in cases 1 and 2 and was not detected in their cord blood. Therefore, the developmental timing of epimutation in these cases is speculated not to be of germ-cell origin, but of post-zygotic origin. Cases 1 and 2 were confirmed to have developed normally at their clinical examination at 3 years old. Case 3 was recorded to be healthy at 6 years old through an interview to its mother.

Both PIH and FGR have been considered to be associated with impaired maternal uteroplacental perfusion secondary to defective extravillous trophoblast invasion [36–38]. *H19* and *IGF2* have been shown to be involved in placental development and trophoblast cell proliferation, differentiation, and migration. In cell culture models, *IGF2* has been shown to prevent apoptosis and enhance proliferation and migration/invasion of human trophoblast cells [reviewed in [39]], differentiation of murine ectoplacental cone trophoblast [40], and migration of ovine trophoblast cells [41]. *H19* has also been shown to promote the migration and invasion of a human extravillous trophoblast cell line [42]. In a mouse model, *H19* and *Igf2* genes are demonstrated to contribute to the control of placental mass and the normal differentiation of giant cells [43]. Disruption of imprinting leading to the biallelic expression of *H19* and repression of *Igf2* is considered to impair placental development and trophoblast differentiation. The placental hypomethylation at *H19*-DMR was associated with PIH in two cases (cases 2 and 3) and with FGR in one case (case 1) in this study. Common pregnancy complications represented by FGR and PIH are heterogeneous conditions for which a variety of genetic and environmental influences can contribute to risk [21]. Therefore, the same epimutation (i.e., placental hypomethylation at *H19*-DMR) can be associated with two different phenotypes, PIH and FGR, depending on the genetic and the environmental factors the fetus and the mother possess and get exposed to.

Despite the efforts by previous and our current studies, many of the findings reported regarding the alteration of DNA methylation at *H19*-DMR are not reproducible among studies. Such inconsistencies seem to originate from various limitations in the studies (including ours), such as sample size, availability of tissues, lack of simultaneous analyses of gene expression, and DNA methylation. To elucidate molecular etiologies of multifactorial pregnancy-associated complications, such as FGR and PIH, where all genetic factors of both mother and baby, environmental factors, and epigenetic perturbations need to be considered, it will be necessary to collect genomic, transcriptomic, and epigenomic data in an unbiased manner from mother and baby (soma and placenta) of normal and abnormal pregnancies at a larger scale than ever before. Clinical follow-up of enrolled individuals is important to assess the effects of placental functions on individual postnatal phenotypes.

## Conclusions

Our study demonstrated that *H19*-DMR epimutation could occur in a placenta-specific manner, presumably post-zygotically, and potentially be associated with pregnancy complications, but at a low frequency (1.5%). Alteration of the *H19/IGF2* expression ratio due to hypomethylation of *H19*-DMR may have been involved in the pathogenesis of pregnancy complications. Further characterization of the *H19/IGF2* imprinted gene cluster in normal pregnancy and pregnancy complication cases at a larger scale and conducted in a more comprehensive manner will help elucidate its involvement with placental development and relevant diseases in humans.

## Methods

### Subjects

This study was approved by the Institutional Review Board Committees at the National Center for Child Health and Development (NCCHD) of Japan, Kyushu University Hospital, and Hokkaido University Hospital, and written informed consent was obtained from all participants. Placenta, corresponding umbilical cord blood, and parental blood were collected for normal, FGR, and PIH pregnancy cases. A total of 140 cases (105 FGR, 12 PIH, and 23 FGR/PIH cases) and 39 controls were obtained from NCCHD and Kyushu University Hospital as case group I and control group I, respectively. A total of 62 cases (27 FGR, 26 PIH, and 9 FGR/PIH cases) and 29 controls were obtained from Hokkaido University Hospital as case group II and control group II, respectively. Ogawa’s birthweight percentile tables [44] were used for the diagnosis of FGR. Singleton newborns at 37–41 weeks of gestation with birthweight > 10th percentile without any complications were enrolled as controls. Pregnancy with birthweight < 10th percentile was diagnosed as FGR (gestational age at birth and sex were considered). Pregnancy with hypertension (sBP > 140 mmHg and/or dBP > 90 mmHg) with or without proteinuria (> 300 mg/day) after 20 weeks was diagnosed as PIH. Newborns with karyotypic abnormality and multiple conceptions were excluded. The mean of gestational ages, fetal and placental weights for case and control groups, and individual data for the cases that exhibited hypomethylation at *H19*-DMR (case 1, case 2, and case 3) are provided in Table 1.

### Sample collection and preparations

We dissected out the chorionic plate from the placentas of case group I and control group I collected at NCCHD and Kyushu University (set I) and chorionic villi from the placentas of case group II and control group II collected at Hokkaido University Hospital (set II). It should be noted that the difference in the tissue types of placenta collected between set I and set II was not rationally planned. We initiated the placental sample collection for set I at NCCHD and chose the chorionic plate as the target tissue. When we sought for an independent set of placental samples from common pregnancy complications and controls, only the sample set of chorionic villi (set II) was available. A piece of chorionic plate tissue was divided into two pieces (one for genomic DNA extraction and the other for RNA extraction) and washed with 1× PBS. Chorionic plate samples for genomic DNA extraction were stored at − 80 °C until genomic DNA extraction. Chorionic plate samples for RNA extraction were soaked in RNAlater (Qiagen) overnight at 4 °C and stored at − 80 °C. Chorionic villi were collected, washed with 1× PBS repeatedly, divided into two tubes, and stored at − 80 °C until genomic DNA and total RNA extraction. Genomic DNA was extracted using either the standard phenol/chloroform extraction method or QIAamp DNA Blood Midi Kit (Qiagen). Total RNA was extracted using TRIzol Reagent (Invitrogen). Complementary DNA (cDNA) synthesis was carried out using QuantiTect Reverse Transcription Kit (Qiagen).

### DNA methylation analyses

For *H19*-DMR_r1, the PCR primers described previously [45] were used in COBRA and pyrosequencing analyses. For *H19*-DMR_r3, a pair of primers that amplify a 256-bp fragment and another pair of primers that amplify a 476-bp fragment were designed for COBRA and pyrosequencing, respectively. A 195-bp interval located in between r1 and r3 regions was designated as *H19*-DMR_r2 and subjected to pyrosequencing analysis. A 352-bp region within *IGF2*-DMR0 [46, 47] and a 337-bp region within *IGF2*-DMR2 [12, 46] were also subjected to pyrosequencing analysis. Bisulfite-PCR primers for COBRA were designed using the MethPrimer (http://www.urogene.org/methprimer/index1.html). Bisulfite-PCR and sequencing primers for pyrosequencing were designed using the PyroMark Assay Design 2.0 (Qiagen). Detailed information for the primers is listed in Additional file 4: Table S2.

One microgram of genomic DNA was bisulfite-treated and purified using the EpiTect Bisulfite Kit (Qiagen). PCR amplification for COBRA assays for *H19*-DMR (subregions r1 and r3) was performed in a total volume of 20 μL containing 20 ng of bisulfite-converted DNA, 20 pmol each primer, dNTPs (2.5 mM each), 1.5 U ExTaq HS (Takara), and supplied buffer. The standard thermal cycling conditions used were as follows: initial denaturation at 94 °C for 5 min, followed by 35 cycles of denaturation at 94 °C for 30 s, annealing for 30 s, and extension at 72 °C for 30 s (the annealing temperature for each primer pair is listed in Additional file 4: Table S2). The PCR products were purified using the Illustra GFX 96 PCR purification kit (GE Healthcare). One fourth of the purified PCR products were digested with 6 U of TaqI (NEB) in a 15-μL scale reaction for 2 h. One out of 15 μL was electrophoresed using the MultiNA Microchip Electrophoresis System and DNA-1000 reagents kit (Shimadzu, Japan). The methylation percent value for each sample was calculated as described previously [48]. COBRA assay conditions for LINE1 and 16 imprinted DMRs other than *H19*-DMR were described previously [49, 50].

Pyrosequencing was carried out using the PyroMarkQ24 system (Qiagen) and PyroMark Gold Q24 Reagents (Qiagen) according to the manufacturer’s instructions. In brief, the biotinylated PCR products were purified with the Streptavidin Sepharose HP beads (GE Healthcare). The purified PCR products were washed, denatured, and then annealed with a sequencing primer. Quantification of methylated cytosine was performed using PyroMark Q24 software (Qiagen).

For bisulfite sequencing, PCR products were cloned using the StrataClone PCR Cloning Kit (Agilent Technologies) and transformed into StrataClone Competent Cells. Single colonies were picked up and used as starting material to amplify plasmid DNA within them using the TempliPhi DNA Amplification Kit (GE Healthcare). Sequencing reactions for individual amplified clones were conducted using the BigDye Terminator version 3.1 Cycle Sequencing kit (Applied Biosystems) with the M13 Rev primer. Sequence data were obtained using ABI3130xl Genetic Analyzer and analyzed using the QUMA website (http://quma.cdb.riken.jp/) [51]. SNPs, rs2107425 (A/G) within the *H19*-DMR_r1, and rs2251312 (C/G) and a T/G SNP at chr11:2,019,694 (hg19) within the *H19*-DMR_r3 were used to distinguish parental alleles.

### Allelic expression analysis for H19

First-strand cDNA was synthesized from 1 μg of placental total RNA using QuantiTect Reverse Transcription Kit (Qiagen). For the allelic expression analysis of *H19*, an exonic SNP rs2067051 (A/G) was used to distinguish parental alleles. RT-PCR was performed in a total volume of 20 μL containing one tenth of the RT reaction, 20 pmol each primer, dNTPs (2.5 mM each), 1.5 U ExTaq HS (Takara), and supplied buffer. The RT-PCR products were treated with ExoSAP- IT (GE Healthcare) and subjected to direct sequencing using one of the PCR primers as a sequencing primer. The details for the primers are listed in Additional file 4: Table S2.

### Quantitative RT-PCR

First-strand cDNAs synthesized using the QuantiTect Reverse Transcription Kit were subjected to real-time PCR analysis using the SYBR Premix Ex Taq (RR420A, Takara) on an ABI7500fast system (Thermo-Fisher Scientific). The thermal cycling conditions used were the initial denaturation at 95 °C for 1 min, followed by the 40 cycles of 95 °C for 15 s and 60 °C for 60 s. The sequences of the PCR primers used are listed in Additional file 4: Table S2. The relative expression levels of *H19* and *IGF2* were calculated by the delta-delta cycle threshold (Ct) method. Delta Ct values were calculated using the Ct values of *ACTB* as the normalization control, and delta-delta Ct values corresponding to relative expression levels were calculated using a placenta sample of normal pregnancy as the reference.

### Chromosomal copy number analysis

HumanCytoSNP-12 BeadChip data were obtained as described previously [52] and were visualized using KaryoStudio Data Analysis Software version 1.4.3.0 Build 37 (Illumina). Copy number analysis was conducted using CNV Plugin V3.0.7.0 (Illumina) with its default parameters for copy number gain/loss and copy neutral loss of heterozygosity.

## Additional files

Additional file 1: **Figure S1.** Correlation plots for DNA methylation levels of *H19*-DMR and *IGF2*-DMRs in the placenta and cord blood samples of case/control set I and in the placenta samples of case/control set II. **Figure S2.** Methylation levels of 18 imprinted DMRs and LINE1 repetitive elements in the placentas of cases 1–3 and controls determined by COBRA assays. (PDF 333 kb)

Additional file 2: **Figure S3.** Absence of copy number alterations at the *H19*-IGF2 imprinted gene cluster in the placenta and cord blood samples of cases 1, 2, and 3. (PDF 395 kb)

Additional file 3: **Table S1.** Mean methylation levels of four CpG sites within H19-DMR and IGF2-DMRs measured by COBRA in case and control groups. (XLSX 13 kb)

Additional file 4: List of primers. (XLSX 12 kb)

## Data Availability

All data generated and analyzed throughout this study are included in this published article and its supplementary information files.

## Abbreviations

DMR: Differentially methylated region
FGR: Fetal growth restriction
HDP: Hypertensive disorder of pregnancy
ICR: Imprinting control region
LINE1: Long interspersed nuclear element 1
LOI: Loss of imprinting
PIH: Pregnancy-induced hypertension
ROI: Relaxation of imprinting

## References

[1] Arnaud P (2010). Genomic imprinting in germ cells: imprints are under control. Reproduction..

[2] Delaval K, Wagschal A, Feil R (2006). Epigenetic deregulation of imprinting in congenital diseases of aberrant growth. Bioessays..

[3] Weksberg R, Shuman C, Beckwith JB (2010). Beckwith-Wiedemann syndrome. Eur J Hum Genet..

[4] Mussa A, Molinatto C, Baldassarre G, Riberi E, Russo S, Larizza L (2016). Cancer risk in Beckwith-Wiedemann syndrome: a systematic review and meta-analysis outlining a novel (epi)genotype specific histotype targeted screening protocol. J Pediatr..

[5] Gicquel C, Rossignol S, Cabrol S, Houang M, Steunou V, Barbu V (2005). Epimutation of the telomeric imprinting center region on chromosome 11p15 in Silver-Russell syndrome. Nat Genet..

[6] Leighton PA, Ingram RS, Eggenschwiler J, Efstratiadis A, Tilghman SM (1995). Disruption of imprinting caused by deletion of the H19 gene region in mice. Nature..

[7] Eggenschwiler J, Ludwig T, Fisher P, Leighton PA, Tilghman SM, Efstratiadis A (1997). Mouse mutant embryos overexpressing IGF-II exhibit phenotypic features of the Beckwith-Wiedemann and Simpson-Golabi-Behmel syndromes. Genes Dev..

[8] Angiolini E, Coan PM, Sandovici I, Iwajomo OH, Peck G, Burton GJ (2011). Developmental adaptations to increased fetal nutrient demand in mouse genetic models of Igf2-mediated overgrowth. FASEB J..

[9] DeChira TM, Efstratiadis A, Robertson EJ (1990). A growth-deficiency phenotype in heterozygous mice carrying an insulin-like growth factor II gene disrupted by targeting. Nature..

[10] Lopez MF, Dikkes P, Zurakowski D, Villa-Komaroff L (1996). Insulin-like growth factor II affects the appearance and glycogen content of glycogen cells in the murine placenta. Endocrinology..

[11] Coan PM, Fowden AL, Constancia M, Ferguson-Smith AC, Burton GJ, Sibley CP (2008). Disproportional effects of Igf2 knockout on placental morphology and diffusional exchange characteristics in the mouse. J Physiol..

[12] Guo L, Choufani S, Ferreira J, Smith A, Chitayat D, Shuman C (2008). Altered gene expression and methylation of the human chromosome 11 imprinted region in small for gestational age (SGA) placentae. Dev Biol..

[13] Diplas AI, Lambertini L, Lee MJ, Sperling R, Lee YL, Wetmur J (2009). Differential expression of imprinted genes in normal and IUGR human placentas. Epigenetics..

[14] Yu L, Chen M, Zhao D, Yi P, Lu L, Han J (2009). The H19 gene imprinting in normal pregnancy and pre-eclampsia. Placenta..

[15] Bourque DK, Avila L, Peñaherrera M, von Dadelszen P, Robinson WP (2010). Decreased placental methylation at the H19/IGF2 imprinting control region is associated with normotensive intrauterine growth restriction but not preeclampsia. Placenta..

[16] Tabano S, Colapietro P, Cetin I, Grati FR, Zanutto S, Mandò C (2010). Epigenetic modulation of the IGF2/H19 imprinted domain in human embryonic and extra-embryonic compartments and its possible role in fetal growth restriction. Epigenetics..

[17] Koukoura O, Sifakis S, Zaravinos A, Apostolidou S, Jones A, Hajiioannou J (2011). Hypomethylation along with increased H19 expression in placentas from pregnancies complicated with fetal growth restriction. Placenta..

[18] Rancourt RC, Harris HR, Barault L, Michels KB (2013). The prevalence of loss of imprinting of H19 and IGF2 at birth. FASEB J..

[19] Mol BWJ, Roberts CT, Thangaratinam S, Magee LA, de Groot CJM, Hofmeyr GJ (2016). Pre-eclampsia. Lancet.

[20] Nardozza LM, Caetano AC, Zamarian AC, Mazzola JB, Silva CP, Marçal VM, Lobo TF, Peixoto AB, Araujo Júnior E (2017). Fetal growth restriction: current knowledge. Arch Gynecol Obstet..

[21] Manokhina I, Del Gobbo GF, Konwar C, Wilson SL, Robinson WP (2017). Review: placental biomarkers for assessing fetal health. Hum Mol Genet..

[22] Takai D, Gonzales FA, Tsai YC, Thayer MJ, Jones PA (2001). Large scale mapping of methylcytosines in CTCF binding sites in the human H19 promoter and aberrant hypomethylation in human bladder cancer. Hum Mol Genet..

[23] Vu TH, Li T, Nguyen D, Nguyen BT, Yao XM, Hu JF (2000). Symmetric and asymmetric DNA methylation in the human IGF2-H19 imprinted region. Genomics..

[24] Lopes S, Lewis A, Hajkova P, Dean W, Oswald J, Forné T (2003). Epigenetic modifications in an imprinting cluster are controlled by a hierarchy of DMRs suggesting long-range chromatin interactions. Hum Mol Genet..

[25] Bell AC, Felsenfeld G (2000). Methylation of a CTCF-dependent boundary controls imprinted expression of the Igf2 gene. Nature..

[26] Singh P, Lee DH, Szabó PE (2012). More than insulator: multiple roles of CTCF at the H19-Igf2 imprinted domain. Front Genet..

[27] Robinson WP, Peñaherrera MS, Jiang R, Avila L, Sloan J, McFadden DE (2010). Assessing the role of placental trisomy in preeclampsia and intrauterine growth restriction. Prenat Diagn..

[28] Wan P, Su W, Zhang Y, Li Z, Deng C, Li J, et al. LncRNA H19 initiates microglial pyroptosis and neuronal death in retinal ischemia/reperfusion injury. Cell Death Differ. 2019; [Epub ahead of print] PubMed PMID: 31127201. 10.1038/s41418-019-0351-4 PMC7206022 31127201

[29] Cao T, Jiang Y, Wang Z, Zhang N, Al-Hendy A, Mamillapalli R, et al. H19 lncRNA identified as a master regulator of genes that drive uterine leiomyomas. Oncogene. 2019; [Epub ahead of print] PubMed PMID: 31089260. 10.1038/s41388-019-0808-4 PMC6755985 31089260

[30] Zhang L, Yang Z, Huang W, Wu J (2019). H19 potentiates let-7 family expression through reducing PTBP1 binding to their precursors in cholestasis. Cell Death Dis..

[31] Zhou J, Xu J, Zhang L, Liu S, Ma Y, Wen X (2019). Combined single-cell profiling of lncRNAs and functional screening reveals that H19 is pivotal for embryonic hematopoietic stem cell development. Cell Stem Cell..

[32] Morita S, Noguchi H, Horii T, Nakabayashi K, Kimura M, Okamura K (2016). Targeted DNA demethylation in vivo using dCas9-peptide repeat and scFv-TET1 catalytic domain fusions. Nat Biotechnol..

[33] Moore GE, Ishida M, Demetriou C, Al-Olabi L, Leon LJ, Thomas AC (2015). The role and interaction of imprinted genes in human fetal growth. Philos Trans R Soc Lond B Biol Sci..

[34] Yamazawa K, Kagami M, Nagai T, Kondoh T, Onigata K, Maeyama K (2008). Molecular and clinical findings and their correlations in Silver-Russell syndrome: implications for a positive role of IGF2 in growth determination and differential imprinting regulation of the IGF2-H19 domain in bodies and placentas. J Mol Med..

[35] Higashimoto K, Nakabayashi K, Yatsuki H, Yoshinaga H, Jozaki K, Okada J (2012). Aberrant methylation of H19-DMR acquired after implantation was dissimilar in soma versus placenta of patients with Beckwith-Wiedemann syndrome. Am J Med Genet A..

[36] Sones JL, Davisson RL (2016). Preeclampsia, of mice and women. Physiol Genomics..

[37] Mifsud W, Sebire NJ (2014). Placental pathology in early-onset and late-onset fetal growth restriction. Fetal Diagn Ther..

[38] Kaufmann P, Black S, Huppertz B (2003). Endovascular trophoblast invasion: implications for the pathogenesis of intrauterine growth retardation and preeclampsia. Biol Reprod..

[39] Sferruzzi-Perri AN, Sandovici I, Constancia M, Fowden AL (2017). Placental phenotype and the insulin-like growth factors: resource allocation to fetal growth. J Physiol..

[40] Kanai-Azuma M, Kanai Y, Kurohmaru M, Sakai S, Hayashi Y (1993). Insulin-like growth factor (IGF)-I stimulates proliferation and migration of mouse ectoplacental cone cells, while IGF-II transforms them into trophoblastic giant cells in vitro. Biol Reprod..

[41] Kim J, Song G, Gao H, Farmer JL, Satterfield MC, Burghardt RC (2008). Insulin-like growth factor II activates phosphatidylinositol 3-kinase-protooncogenic protein kinase 1 and mitogen-activated protein kinase cell Signaling pathways, and stimulates migration of ovine trophectoderm cells. Endocrinology..

[42] Zuckerwise L, Li J, Lu L, Men Y, Geng T, Buhimschi CS (2016). H19 long noncoding RNA alters trophoblast cell migration and invasion by regulating TβR3 in placentae with fetal growth restriction. Oncotarget..

[43] Kawahara M, Wu Q, Yaguchi Y, Ferguson-Smith AC, Kono T (2006). Complementary roles of genes regulated by two paternally methylated imprinted regions on chromosomes 7 and 12 in mouse placentation. Hum Mol Genet..

[44] Ogawa Y, Iwamura T, Kuriya K, Nishida H, Takeuchi H, Takeda M (1998). Birth size standards by gestational age for Japanese neonates. Acta neonat Jap..

[45] Sasaki K, Soejima H, Higashimoto K, Yatsuki H, Ohashi H, Yakabe S (2007). Japanese and North American/European patients with Beckwith-Wiedemann syndrome have different frequencies of some epigenetic and genetic alterations. Eur J Hum Genet..

[46] Moore T, Constancia M, Zubair M, Bailleul B, Feil R, Sasaki H (1997). Multiple imprinted sense and antisense transcripts, differential methylation and tandem repeats in a putative imprinting control region upstream of mouse Igf2. Proc Natl Acad Sci U S A..

[47] Murrell A, Ito Y, Verde G, Huddleston J, Woodfine K, Silengo MC, Spreafico F (2008). Distinct methylation changes at the IGF2-H19 locus in congenital growth disorders and cancer. PLoS One..

[48] Brena RM, Auer H, Kornacker K, Hackanson B, Raval A, Byrd JC (2006). Accurate quantification of DNA methylation using combined bisulfite restriction analysis coupled with the Agilent 2100 Bioanalyzer platform. Nucleic Acids Res..

[49] Yang AS, Estécio MR, Doshi K, Kondo Y, Tajara EH, Issa JP (2004). A simple method for estimating global DNA methylation using bisulfite PCR of repetitive DNA elements. Nucleic Acids Res..

[50] Yamazawa K, Nakabayashi K, Kagami M, Sato T, Saitoh S, Horikawa R (2010). Parthenogenetic chimaerism/mosaicism with a Silver-Russell syndrome-like phenotype. J Med Genet..

[51] Kumaki Y, Oda M, Okano M (2008). QUMA: quantification tool for methylation analysis. Nucleic Acids Res..

[52] Miyata T, Sonoda K, Tomikawa J, Tayama C, Okamura K, Maehara K (2015). Genomic, epigenomic, and transcriptomic profiling towards identifying omics features and specific biomarkers that distinguish uterine leiomyosarcoma and leiomyoma at molecular levels. Sarcoma..

