# Supplementary material for: Placenta-specific epimutation at H19-DMR among common pregnancy complications: its frequency and effect on the expression patterns of H19 and IGF2
Source: Clin Epigenetics. 2019 Aug 1;11:113. doi: 10.1186/s13148-019-0712-3 (PMC6676526; doi:10.1186/s13148-019-0712-3)
Supplement: Supplementary file 1 — Figure S1. Correlation plots for DNA methylation levels of H19-DMR and IGF2-DMRs in the placenta and cord blood samples of case/control set I and in the placenta samples of case/control set II. Figure S2. Methylation levels of 18 imprinted DMRs and LINE1 repetitive elements in the placentas of cases 1–3 and controls determined by COBRA assays. (PDF 333 kb) [file 13148_2019_712_MOESM1_ESM.pdf]

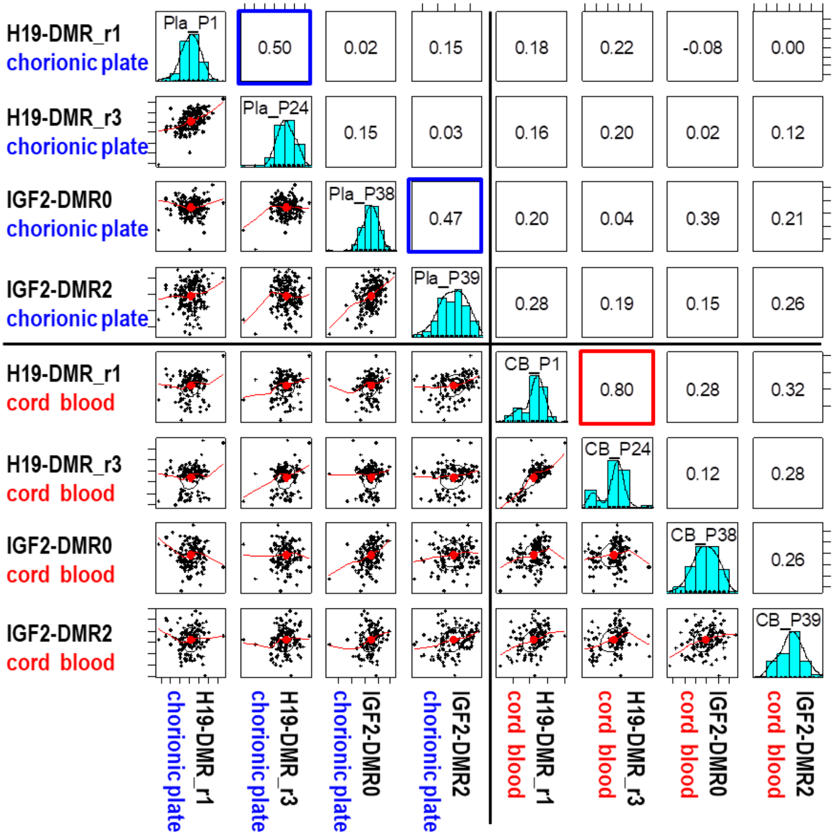

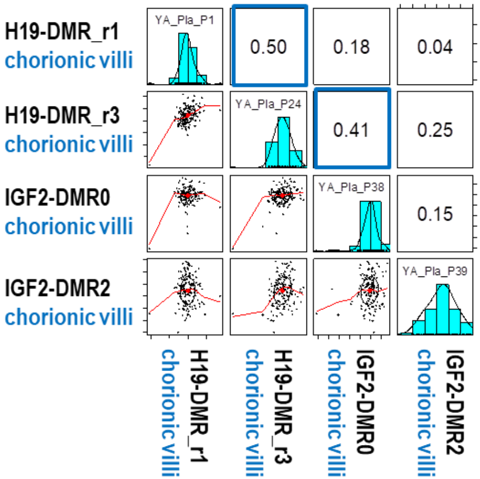

Fig. S1 Yamaguchi *et al.*

Fig. S1: Correlation plots for DNA methylation levels of *H19*-DMR and *IGF2*-DMRs in the placenta and cord blood samples of case/control Set I (top) and in the placenta samples of case/control Set II (bottom). The plots were drawn using the *pairs.panels* function in the *psych* package (R version 3.4.4). Pla and CB indicate placenta and cord blood, respectively. P1, P24, P38, and P39 correspond to bisulfite PCR primer pairs for *H19*-DMR_r1, *H19*-DMR_r3, *IGF2*-DMR0, and *IGF2*-DMR2, respectively. We also performed the same type of analyses only for cases and for controls, and obtained similar results.

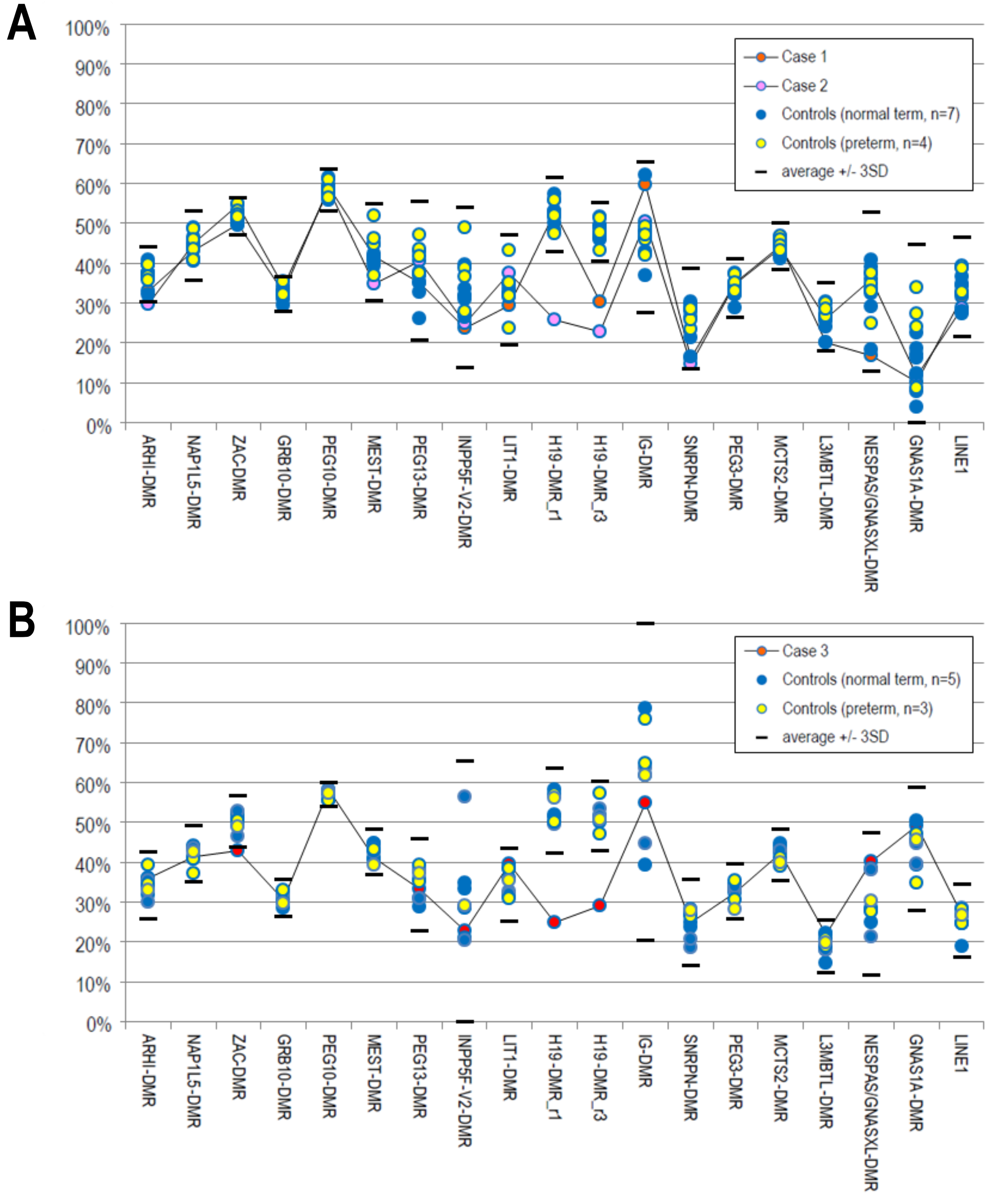

Fig. S2 Yamaguchi *et al.*

Fig. S2: Methylation levels of 18 imprinted DMRs and LINE1 repetitive elements in the placentas of Cases 1-3 and controls determined by COBRA assays.

The methylation levels of two subregions (r1 and r3) within *H19*-DMR, 16 other imprinted DMRs, and LINE1 repetitive elements were measured for chorionic plate of Case 1, Case 2, and controls (**A**), and for chorion of Case 3 and controls (**B**). Methylation levels corresponding to ±3SD of the mean of control samples are shown by horizontal bars. The control samples analyzed are: seven normal-term (37, 37, 38, 39, 39, 40, and 40 weeks) and four preterm (24, 31, 34, and 34 weeks) chorionic plate samples (**A**), and five normal-term (38, 39, 40, 40, and 40 weeks) and three-preterm (28, 32, and 33 weeks) chorion samples (**B**).
