# Supplementary material for: Placenta-specific epimutation at H19-DMR among common pregnancy complications: its frequency and effect on the expression patterns of H19 and IGF2
Source: Clin Epigenetics. 2019 Aug 1;11:113. doi: 10.1186/s13148-019-0712-3 (PMC6676526; doi:10.1186/s13148-019-0712-3)
Supplement: Supplementary file 2 — Figure S3. Absence of copy number alterations at the H19-IGF2 imprinted gene cluster in the placenta and cord blood samples of cases 1, 2, and 3. (PDF 395 kb) [file 13148_2019_712_MOESM2_ESM.pdf]

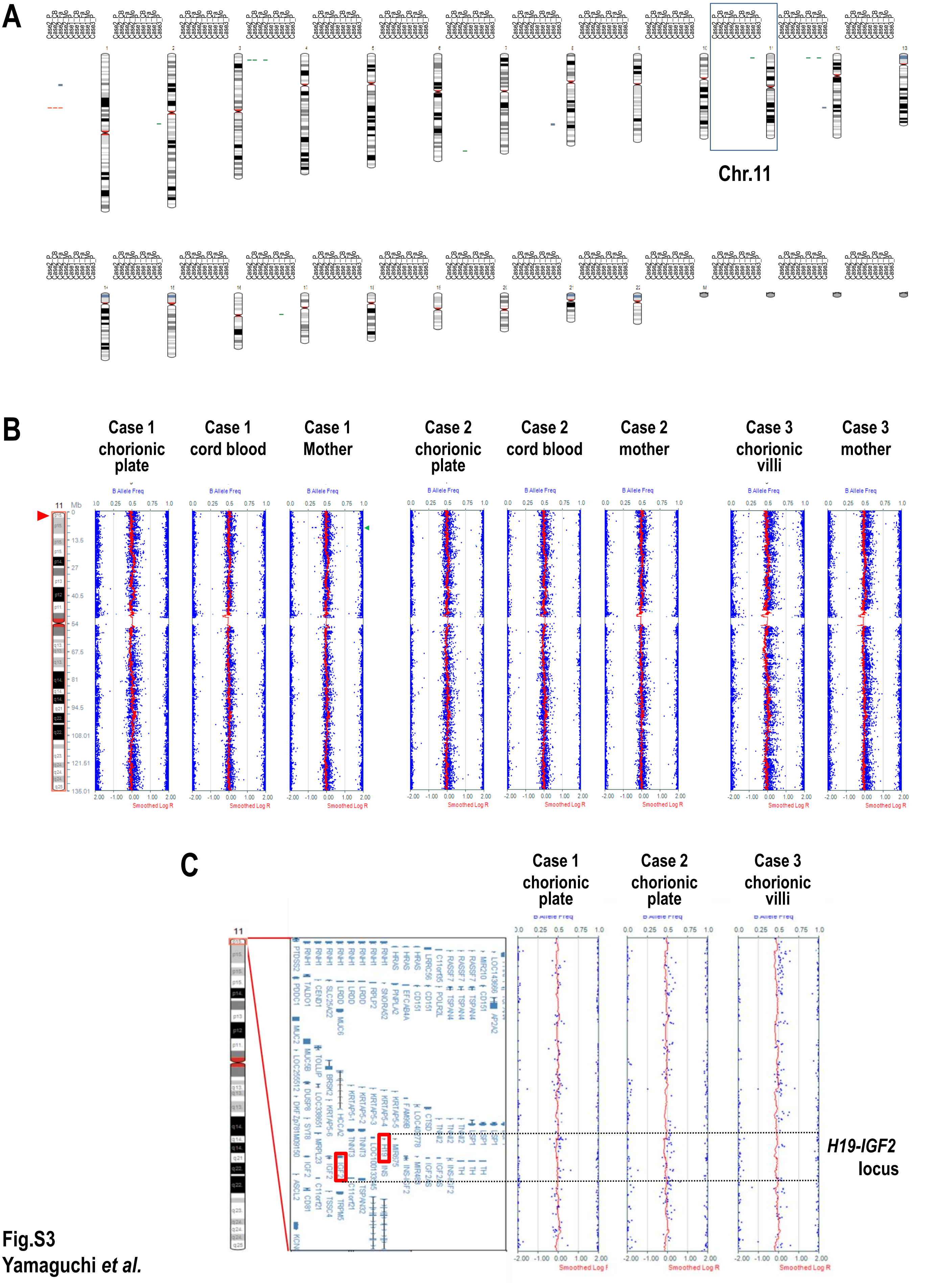

Fig.S3
Yamaguchi *et al.*

Fig.S3: Absence of copy number alterations at the *H19*-IGF2 imprinted gene cluster in the placenta and cord blood samples of Cases 1, 2, and 3.

Chromosomal copy number alterations were assessed for Cases 1, 2, and 3 and their parents: chorionic plate (P) and cord blood (CB) of Cases 1 and 2 and peripheral blood of their mother (Mo) and father (Fa), and chorionic villi (P) of Case 3 and peripheral blood of Case 3' mother (Mo).

Copy number alterations detected in autosomal chromosomes (**A**). Gain (orange), loss (green), and copy neutral loss of heterozygosity (grey) detected by CNV Plugin V3.0.7.0 are shown as horizontal bars. On the chromosome 11, a three copy region spanning the 172 kb interval of chr11:7,922,651-8,095,414 (hg19) was detected in the mother of Case 1.

B allele frequency (BAF) and log R ratio (LRR) plots for chromosome 11 for selected eight samples (**B**). BAFs (0 to 1.0) are shown in blue dots for each of the SNP probes. Smoothed LRR line plots are shown in red (-2.0 to 2.0). Two copy regions show the BAF pattern of three clusters (0, 0.5, and 1) and the LRR value of 0. The approximate position of *H19-IGF2* cluster locus within the chromosome 11p15.5 band is shown by the red arrow head. The position of the three copy region detected in the mother of Case 1 is shown by the green arrow head.

BAF and LRR patterns at a region containing H19 and IGF2 genes in the placenta samples of Cases 1, 2, and 3 (**C**). As shown, copy number alterations were not detected at the *H19-IGF2* locus in these samples as well as cord blood and parental samples (data not shown).
